# Promoting healthy sexual practices to prevent youth pregnancy in Vhembe district

**DOI:** 10.4102/curationis.v48i1.2711

**Published:** 2025-08-07

**Authors:** Ntiyiso Vinny Khosa, Azwinndini Gladys Mudau, Lufuno Makhado

**Affiliations:** 1 Department of Public Health, Faculty of Health Sciences, University of Venda, Polokwane, South Africa; 2 Department of Public Health, Faculty of Medicine and Health Sciences, Walter Sisulu University, Mthatha, South Africa

**Keywords:** healthy sexual practices, promotion, termination, unplanned pregnancy, youth

## Abstract

**Background:**

Termination of pregnancy has a negative health impact on the lives of youth. Myth and illegal consultation on the termination of pregnancy from unprofessional healthcare personnel, which results in negative consequences on the health and future of youth.

**Objectives:**

The purpose of this study was to examine healthy sexual practices to lessen the termination of unplanned pregnancies among youth in the Vhembe district.

**Method:**

A quantitative survey method was used in this study. A stratified random sampling was used to select sample size of 531 participants. Self-structured questionnaires and the SurveyMonkey were used to collect data. Pre-test was used to check the reliability of the instrument in the study. Data were analysed using Stata version 18. The approval to conduct the study was received from the University of Venda and the Department of Basic Education.

**Results:**

The findings showed that 56% of the participants agreed that the average waiting period at the public health facility is exceedingly long for pregnancy termination. 53.9% of the participants agreed that language used to give instructions by healthcare regarding the termination of pregnancy is a barrier. 48.8% of the participants agreed that the lack of pregnancy termination resources deterred youth from visiting health facilities.

**Conclusion:**

Promoting healthy sexual practices among youth is vital in lessening the termination of unplanned pregnancies. Education on contraception use, abstinence and open communication between partners can help young people make informed decisions about their sexual health and prevent unintended consequences.

**Contribution:**

The study illustrates a good piece of innovative work in health promotion in disadvantaged communities in Vhembe district.

## Introduction

Termination of unplanned pregnancies and sexually transmitted infections (STIs) among youth is a public health issue (Up 2017). Worldwide, illegal abortion is associated with maternal deaths, which constitute 80%, and it is a public health concern (Fatusi et al. 2021). Illegal abortion is elevated in countries that enforce abortion laws, and about 97% of illegal terminations of pregnancies in low-income and middle-income countries (LMICs) nearly all terminations linked with deaths (Matilda 2023). Africa is reported to have an elevated risk of abortion linked to deaths worldwide (Ganatra et al. 2017). Yokoe et al. (2019) reported that induced terminating pregnancy is common across the world. Inadequate knowledge of healthy sexual practices is the main cause of unplanned pregnancies and termination of pregnancy (Ujang & Sutan 2018). Youth are more likely to experience complications, such as septicaemia, internal organ damage, tetanus, sterility, severe vaginal bleeding, incomplete abortion, septic abortion, ill health, infertility and death and life-long conditions and disabilities such as obstetric fistula (Ajayi et al. 2020).

The extensive termination responses appear as a result of ineffective use or non-use of contraceptives (Kayembe 2022). Lebese et al. (2013) reported that socio-economic factors are essential drivers of unplanned pregnancies and the failure that led young women not to make an informed decision, which results in termination of pregnancy. Wunderlich (2020) banned pregnancy termination without recognising any valid reasons. In 2017, about 42% of sexually active youth live in 125 countries where terminations of pregnancy are restrained altogether to protect the health of women (Lavelanet et al. 2018). In South Africa, the National Health Department executed and initiated the clinical guidelines and standards that approved the provision of legal abortion known as Choice on Termination of Pregnancy (CTOP) (Mothiba, Muthelo & Mabaso 2020).

Efforts to combat youth pregnancies have met with limited success. Section 2 (1) (a) of the *CTOP Act*, 92 of 1996 protects the right of persons to make informed decisions regarding reproduction and security as well as control over their bodies (Harries & Constant 2020). The Act also makes stipulations for access to reproductive healthcare services, including contraception, TOP, sexuality education and counselling programmes and services. However, it is stressed that TOP is not a form of contraception or population control (Khoza 2019). As contraceptives are available free of charge, there is an inconsistency between the current large numbers of teen pregnancies and requests for TOPS. Therefore, the ideal situation of unplanned pregnancies is avoided by the effective utilisation of contraceptives (Uhawenimana et al. 2024).

The South African government established several non-governmental organisations (NGOs) and organisations for the provision of healthy sexual practices through awareness programmes to prevent the extent of unplanned pregnancies, STIs and illegal abortions (Vinny, Gladys & Lufuno 2024). However, Homela and Chinyoka (2023) outlined factors that prevent youth from accessing sexual reproductive health and practicing healthy sexual practices for both information and services. This includes a lack of access to suitable information on healthy sexual practices and sexual and reproductive health (SRH) services, which are some of the factors that can expose youth (Favier, Greenberg & Stevens 2018). Creation of awareness campaigns on SRH-related concerns with the contribution of both private and public sectors and approaching youth in a responsible manner can yield positive outcomes (Vinny et al. 2024). Netshikweta, Olaniyi and Tshitangano (2018) reported that youth had misconceptions about healthy sexual practices. Hence, they opt not to access and to use contraception because of myths such as weight gain, infertility and other health problems. This evidence clearly implies that the majority of youth do not use condoms to protect themselves against contracting STIs and human immunodeficiency virus (HIV) (Fakudze 2018).

However, despite the SA government’s efforts to prevent illegal termination of pregnancies among youth aged 15 or younger in the RSA, the number of youth requesting TOPs increases annually (Ramakuela et al. 2016). An estimated 260 000 terminations of pregnancy take place in South Africa every year among youth (Ngoveni 2022). A total of 1200 pregnancies were terminated at a district hospital in Limpopo province between 2017 and 2018 (Barron et al. 2023). Studies in South Africa have testified that risk factors associated with the termination of pregnancy, such as financial problems, being poorly educated, being young, unemployed, dependent on parents, being single and other related problems, were most common (Alemayehu et al. 2017; Bearak et al. 2018; Chae et al. 2017). A study conducted at Giyani under Mopani District reported an elevated proportion of unplanned pregnancies among women who are HIV positive; they like to terminate the pregnancy, and they opt not to terminate because of fear of being misjudged (Ngoveni 2022). Termination of pregnancy cannot be neglected; therefore, there is a necessity for this paper to be conducted. The goal of this paper was to determine healthy sexual practices to lessen the termination of unplanned pregnancies among youth in the Vhembe district.

## Research methods and design

### Study design

The study was a quantitative design, using a survey method to examine healthy sexual practices to lessen the termination of unplanned pregnancy among youth in the Vhembe district (Creswell & Creswell 2017). This method and design were used in the study as they allow variables to be encoded and computerised at the exact time (Tiala 2022). The survey method allows investigators to collect data at a convenient time or data-gathering, and it is low cost.

### Study settings

The study was conducted in Vhembe district in Limpopo Province, South Africa. The district has one regional hospital, one specialised psychiatric hospital, six district hospitals, eight community health centres, 115 clinics and 19 mobile clinics that offer sexual reproductive health services and are user friendly for youth (Mudzusi, Munzhedzi & Mahole 2024). Seven thousand four hundred and seventy youth headed the household in the district, while 56.2% share below the lower poverty line. And 15.2% of youth are unemployed in the Vhembe district.

### Population and sampling

The target population in this study is learners from the selected school of Vhembe district, Limpopo province. The researcher recruited participants in selected schools. Arrangements for data collection were made with the head of schools (principals). The researcher and school principals arranged the venue, which was used during data collection. The researcher introduces the study and the benefits of participating in the study to the study participants. All grades in the selected schools were purposively recruited to participate in the study. Non-probability, purposive sampling technique was used to select the 16 circuits in the Vhembe district (Creswell & Creswell 2017).

#### Sampling procedure of participants

A stratified sampling technique as explained by Turner (2020) was used to select participants in this study from various circuits in the selected schools. The grades in schools are treated as a group of learners known as strata or called strata. Samples are independently made across each stratum using a simple random technique (Mweshi & Sakyi 2020). A simple random technique was used to select participants, using the folding of small papers written ‘yes’ or ‘no’ (Creswell & Creswell 2017). Folded papers were inserted in a jug and mixed by a neutral person (Creswell & Creswell 2017). Those who picked yes formed part of the study, and those who picked no did not form part of the study (Mweshi & Sakyi 2020). Therefore, the sample size of the study was 531 participants who were recruited to participate in the study.

### Instrument and collection

A self-structured administered questionnaire was used to collect data and capture variables from March 2023 to July 2023 using SurveyMonkey, an online survey tool that allows participants to respond online. Arrangements for data collection were arranged with the school principals and heads. The surveys were distributed to learners via Survey Monkey in the form of links. Questionnaires consisted of five sections completed by learners. Consent forms and assent forms were completed by learners before the commencement of data collection, those whose consent and assent forms were not completed did not participate in this study.

### Data analysis

A Stata version 18 was used for data management and analysis. The do file data set was created for data cleaning and coding in preparation for data analysis. A Microsoft Excel program was used to create charts.

### Ethical considerations

Ethical clearance was attained from the University of Venda Human Research Ethics Committee (HREC), (project number: FHS/22/PH/0303). Permission for data collection was granted by the Premier’s office in Limpopo province, the Department of Education as well as the circuit, to have access to the school and administer questionnaires to learners for data collection. Data were treated as confidential, and participants’ names were anonymised.

## Results

Data were collected from the ongoing school youth in Vhembe district. 531 learners participated in this study from different circuits in the district.

### Demographic characteristics

A total number of 531 learners participated in the study with an average age range of 10–25 years. Table 1 presents summaries of the demographic characteristics of learners who responded to the structured questionnaires. The results indicate that 74.0% of the participants were in the age group of 16 to 20 years, 16.4% were 10 to 15 years and the minority 9.6% were aged between 21 and 25 years. For marital status, the results indicate that 100% of participants have never married. For gender, the results indicated that 60.8% were female learners and 39.2% were male learners. For having children, 22% of participants had one child, and 3.6% had two children, which constituted 25.6%. For religion, the results of respondents indicated that 86.3% were Christian, 7.1 belonged to traditional religion, 5.1% belonged to other religions and minority 1.1% belonged to the Islamic religion.

**TABLE 1:** Summaries of the demographic characteristics of learners.

Variable	Frequency	%
**Age (years)**		
10–15	87	16.4
16–20	393	74.0
20–25	51	9.6
**Marital status**		
Never married	531	100.0
Living together	0	0.0
Divorced	0	0.0
**Gender**		
Male	208	39.2
Female	323	60.8
**Having children**		
Yes	136	25.6
No	395	74.4
**How many**		
One	117	22
Two	19	3.6
**Religion**		
Christianity	458	86.3
Traditional	40	7.5
Islam	40	7.5
Other	6	1.1

*Source:* Adapted from Khosa, N.V., 2024, ‘An intervention programme to promote healthy sexual practices among youth in Vhembe District, Limpopo Province’, PhD Thesis, Faculty of Health Sciences, University of Venda, Thohoyandou

### Knowledge of youth about the termination of pregnancy

The percentages of the Likert scale are combined, and the summaries are provided. Table 2 shows the knowledge of youth regarding termination of pregnancy (*n* = 531). The findings showed that 225 (42.3%) of the participants disagreed, while 173 (35.6%) of the participants agreed that illegal abortion is when they terminate pregnancy in health facilities. However, 133 (25.0%) of the participants were not sure whether illegal abortion is when they terminate a pregnancy in the health facilities. The findings showed that 270 (50.9%) of the participants disagreed, while 158 (29.7%) of the participants agreed that legal abortion is when they terminate their pregnancies at the sangoma (traditional healer). However, 104 (19.4%) of the participants were not sure whether legal abortion is when they terminate their pregnancies at the sangoma (traditional healer). The findings showed that 283 (53.3%) of the participants agreed, while 123 (23.2%) of the participants disagreed that legal abortion is when they terminate their pregnancy at health facilities by health professionals. However, 125 (23.5%) of the participants were not sure whether legal abortion is when you terminate a pregnancy at health facilities by health professionals. The findings showed that 325 (63.1%) of the respondents agreed, while 117 (22%) of the respondents disagreed that they feel more comfortable when consulting in private healthcare than in public hospitals for termination of pregnancy. However, 89 (16%) of the respondents were not sure whether they feel more comfortable when consulting in private healthcare than in public hospitals for termination of pregnancy.

**TABLE 2:** The knowledge of youth regarding healthy sexual practices.

Variable	Frequency	%
**Illegal abortion is when you terminate pregnancy in health facilities**		
Agree	81	15.4
Strongly disagree	92	17.3
Not sure	133	25.0
Disagree	108	20.3
Strongly disagree	117	22.0
**Legal abortion is when you terminate a pregnancy at the sangoma (traditional healer)**		
Agree	84	15.8
Strongly disagree	74	13.9
Not sure	103	19.4
Disagree	130	24.5
Strongly disagree	140	26.4
**Legal abortion is when you terminate a pregnancy at health facilities by health professionals**		
Agree	143	26.9
Strongly disagree	140	26.4
Not sure	125	23.5
Disagree	59	11.1
Strongly disagree	64	12.1
**I feel more comfortable when consulting in private healthcare than in public hospital for termination of pregnancy**		
Agree	214	40.3
Strongly disagree	111	20.9
Not sure	89	16.8
Disagree	59	11.1
Strongly disagree	58	10.9

*Source:* Adapted from Khosa, N.V., 2024, ‘An intervention programme to promote healthy sexual practices among youth in Vhembe District, Limpopo Province’, PhD Thesis, Faculty of Health Sciences, University of Venda, Thohoyandou

### Healthcare professionals should explain the consequences of pregnancy termination

**FIGURE 1:**
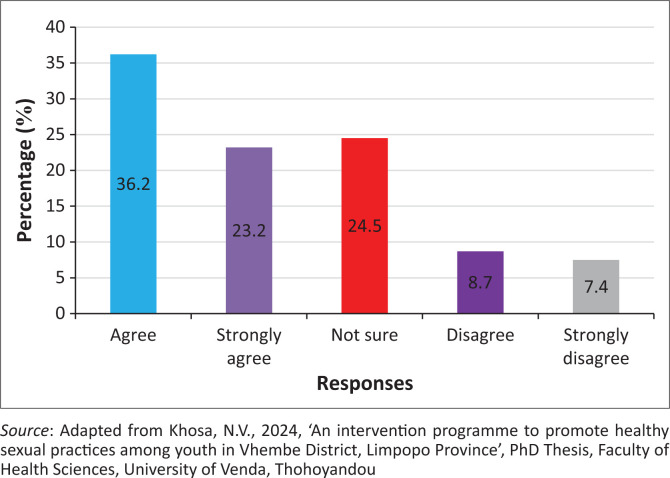
Healthcare professionals should explain the consequences of pregnancy termination.

The findings showed that 315 (59.4%) of the participants agreed, while 86 (16.2%) of the participants disagreed that the healthcare professionals should explain the consequences of pregnancy termination. However, 130 (24.5%) of the participants were neutral on whether healthcare professionals should explain the consequences of pregnancy termination (Figure 1).

### Healthcare staff do not respect youth during their consultation for the termination of pregnancy

**FIGURE 2:**
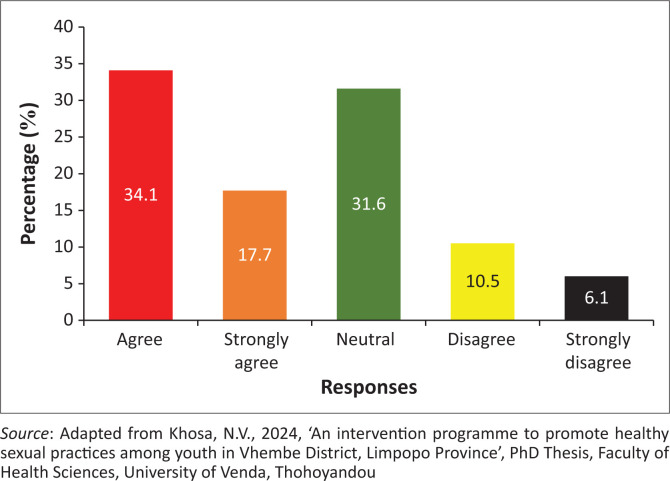
Healthcare staff do not respect youth during their consultation for the termination of pregnancy.

The findings showed that 275 (51.8%) of the participants agreed, while 88 (16.6%) of the participants disagreed that healthcare staff do not respect youth during consultation for termination of pregnancy. However, 168 (31.6%) of the participants were neutral on whether healthcare staff did not respect youth during consultation for termination of pregnancy (Figure 2).

### Health practitioners do not keep the confidentiality of youth accessing health facilities for pregnancy termination

**FIGURE 3:**
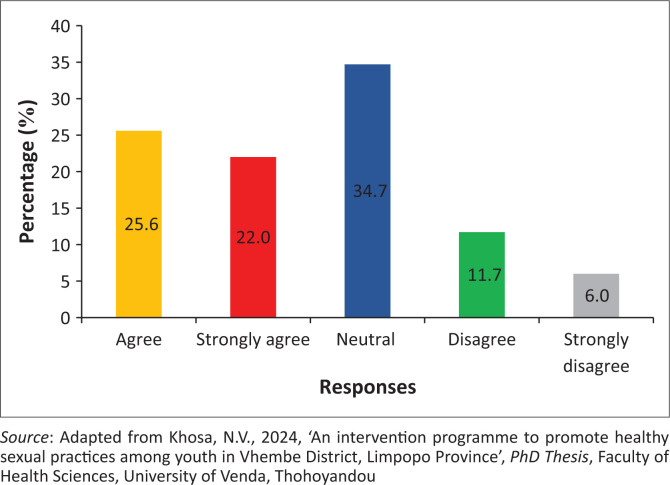
Health practitioners do not keep the confidentiality of youth accessing health facilities for pregnancy termination.

The findings showed that 253 (47.6%) of the participants agreed, while 94 (17.7%) of the participants disagreed that health practitioners do not keep the confidentiality of youth accessing health facilities for pregnancy termination. However, 35 (10.3%) of the participants were neutral on whether health practitioners should keep the confidentiality of youth accessing health facilities for pregnancy termination (Figure 3).

### Language is a barrier when healthcare professionals give instructions regarding the termination of pregnancy

**FIGURE 4:**
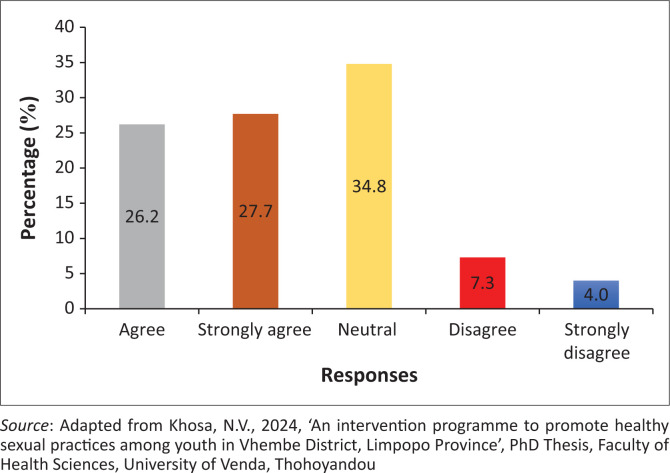
Language is a barrier when healthcare professionals give instruction regarding termination of pregnancy.

The findings showed that 286 (53.9%) of the participants agreed, while 60 (11.3%) of the participants disagreed that the language used to give instructions by healthcare regarding the termination of pregnancy is a barrier. However, 185 (34.8%) of the participants were neutral on whether the language used to give instructions by healthcare regarding the termination of pregnancy is a barrier (Figure 4).

### Health practitioners wanted consent from their parents before they terminated their pregnancy

**FIGURE 5:**
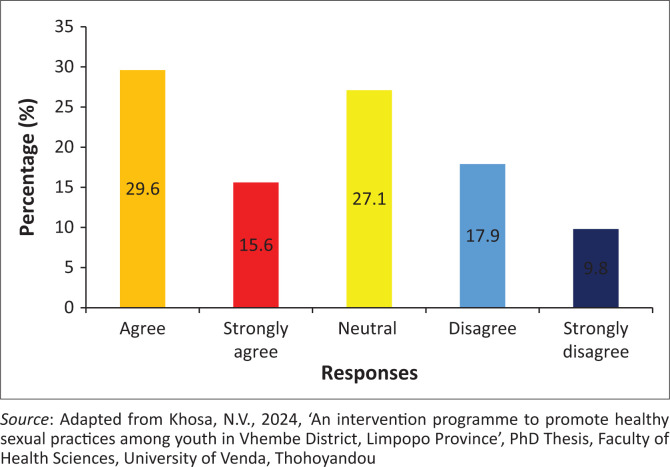
Health practitioners wanted consent from their parents before they terminated their pregnancy.

The findings showed that 240 (45.2%) of the participants agreed, while 144 (27.7%) of the participants disagreed that health practitioners wanted consent from their parents before they terminated their pregnancy. However, 144 (27.1%) of the participants were neutral whether health practitioners wanted consent from their parents before they terminated my pregnancy (Figure 5).

### Health practitioner informed their parent about the consultation they made regarding the termination of pregnancy

**FIGURE 6:**
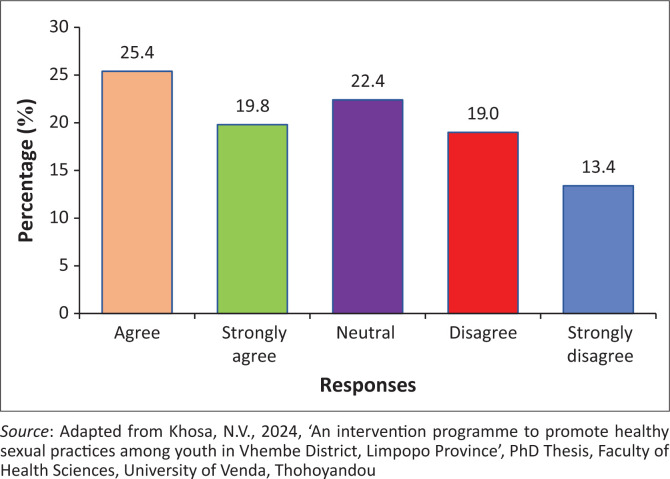
Health practitioner informed their parent about the consultation they made regarding the termination of pregnancy.

The findings showed that 240 (45.2%) of the participants agreed, while 172 (32.4%) of the participants disagreed that health practitioner informed their parents about the consultation they made regarding termination of pregnancy. However, 119 (22.4%) of the participants were neutral on whether health practitioner informed their parents about the consultation they made regarding the termination of pregnancy (Figure 6).

### Lack of pregnancy termination resources deterred youth from visiting a health facility

**FIGURE 7:**
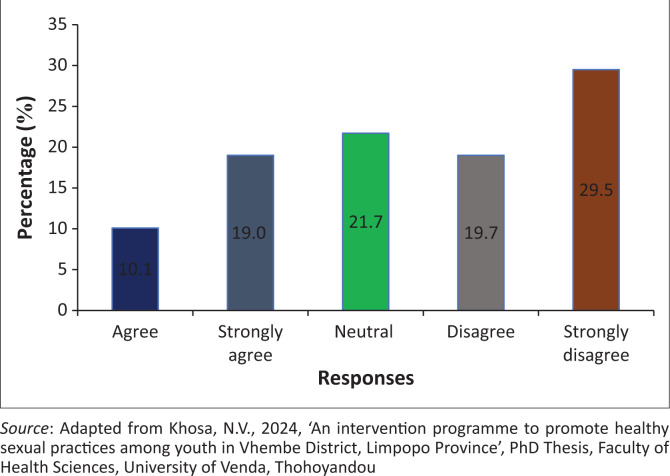
Lack of pregnancy termination resources deterred youth from visiting a health facility.

The findings showed that 259 (48.8%) of the participants agreed, while 157 (29.5%) of the participants disagreed that the lack of pregnancy termination resources deterred youth from visiting health facilities. However, 115 (21.7%) of the participants were neutral on whether the lack of pregnancy termination resources deterred youth from visiting health facilities (Figure 7).

### The average waiting period at the public health facility is very long for pregnancy termination

**FIGURE 8:**
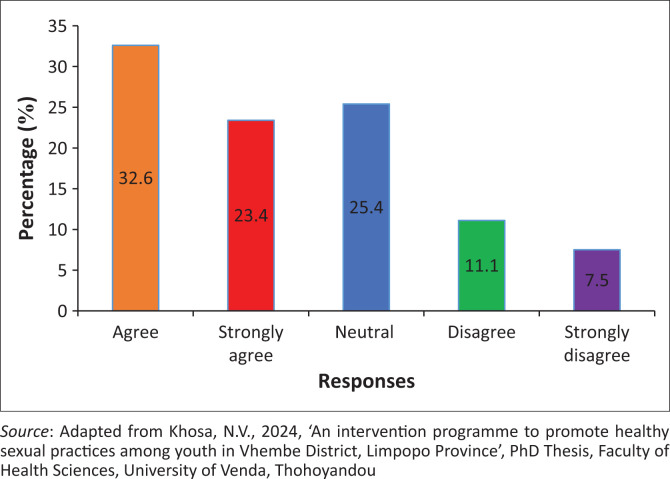
The average waiting period at the public health facility is very long for pregnancy termination.

The findings showed that 297 (56%) of the participants agreed, while 99 (18.6%) of the participants disagreed that the average waiting period at the public health facility is very long for pregnancy termination. However, 135 (25.4%) of the participants were neutral on whether the average waiting period at the public health facility is very long for pregnancy termination (Figure 8).

## Discussion

The first vital finding in this study is the promotion of healthy sexual practices among youth in order to decrease the termination of pregnancies. The findings reveal that illegal abortion is when you terminate pregnancy in health facilities. It was observed that a lack of accurate knowledge about illegal abortion is a detrimental concern among youth (Suh & McReynolds-Pérez 2023). It was reported that the youth had a fear of visiting the health facilities regarding the termination of pregnancy. However, they prefer traditional mixed herbs from unprofessional individuals (Xing et al. 2023). Getahun et al. (2023) emphasise that youth should be taught accurate information that safe abortion is performed in health facilities and requires no payments. It was attested that youth must be provided with accurate information when visiting health facilities for consultation; this will reduce the negative health impact caused by illegal abortion, such as removal of the uterus and excessive bleeding (Xing et al. 2023).

### Legal abortion is when you terminate a pregnancy at the sangoma (traditional healer)

The findings reveal that legal abortion is when you terminate a pregnancy at the sangoma (traditional healer). Supporting findings indicate that youth do not know how to distinguish illegal abortion and legal abortion (Geldenhuys 2019). They rate sangoma and health professionals similarly. Contrary findings reveal that abortion from traditional healers has negative health effects because of the toxification of mixed herbs (Sachs 2023). As a result, traditional healers are unable to measure their medication so that it does not harm the womb and cause excessive bleeding. Contrary findings reveal that when patients have excessive bleeding at the health facilities, they are able to provide blood donors; they regularly monitor patients who performed an abortion (Moloisane-Ledwaba 2022). A study conducted in Johannesburg Central revealed that the majority of the traditional healers who offer abortion are not qualified sangoma, they fly by night and they are not registered with the sangoma regulatory body (Counsellor) (Geldenhuys 2019). It was suggested that traditional healers who perform abortions must be prosecuted as it results in complications and negative health impacts on the future of youth (Maluleke 2022). As a result of abortion from traditional healers, they are unable to conceive pregnancy.

### Legal abortion is when you terminate a pregnancy at health facilities by health professionals

The findings reveal that legal abortion is when you terminate a pregnancy at health facilities by health professionals. Safe abortion is performed in health facilities by health professionals (Skuster, Moseson & Perritt 2023). It was attested that safe medications are used for abortion during termination in the hospitals (Moloisane-Ledwaba 2022). The hospital further monitors their patients after the termination of pregnancy. Contrary findings reveal that health workers are negligent patients after the termination of pregnancy (Sachs 2023). Similar findings indicate that nurses are rude when giving instructions during the termination of pregnancy processes (Getahun et al. 2023). This discourages youth from performing termination of pregnancy in health facilities. It was stated that health professionals are impatient towards youth during the termination of pregnancy; this attitude and behaviour discourage youth from performing termination of pregnancy in the health facilities (Skuster et al. 2023). As a result, it was revealed that patients were turned into jokes during the consultation by emphasising that they were not there when the patient had sexual intercourse. This type of behaviour and attitude makes them unprofessional, and it discourages youth from consulting for termination of pregnancy at the hospital (Geldenhuys 2019).

### They feel more comfortable when consulting in private healthcare than in public hospitals for termination of pregnancy

The findings reveal that they feel more comfortable when consulting in private healthcare than in public hospitals for termination of pregnancy. Concurred findings indicate that private health workers are too welcoming when consulting in their institution (Cleetus et al. 2022). Their services are of high quality, patients do not wait too long in the queues when compared to the public sector hospitals (Cleetus et al. 2022). Contrary findings reveal that private-sector hospitals require upfront payments before consultations; however, public-sector hospitals offer services without payments (Maluleke 2022). Payments will be made later when they have money. This disadvantages youth from disadvantaged backgrounds from visiting private sectors for consultation (Sachs 2023).

### Healthcare professionals should explain the consequences of pregnancy termination

The findings reveal that healthcare professionals should explain the consequences of pregnancy termination. It was affirmed that healthcare professionals should provide appropriate information about the termination of pregnancy (Stifani et al. 2022). Youths should be notified about the negative consequences of abortion from either a hospital or sangoma (Bourke 2022). It was alluded that hospitals must establish programmes to educate youth about the consequences of termination of abortions (Stifani et al. 2022). This will result in a reduction of illegal abortions and their consequences among youth. A contended finding reveals that youth prefer their own choice when it comes to the termination of pregnancy. Katz et al. (2022) urged that this choice be undertaken under the fear and supervision of parents; hence, they perform abortions in traditional healers. Communication between parent and their children is required to enable children to reveal when they are pregnant to their parents before they perform illegal abortions (Katz et al. 2022).

### Healthcare staff do not respect youth during their consultation for the termination of pregnancy

The findings reveal that healthcare staff do not respect them during consultation for the termination of pregnancy. Nurses leave patients unattended by showing that they are at lunch (Wellington, Hegarty & Tarzia 2021). Wellington et al. (2021) attested that nurses do not have tender care towards consulting patients regarding the termination of pregnancy. Coinciding findings by Teffo and Rispel (2020) stated that nurses were rude during the consultations and did not respect the *POPIA Act* of the health institutions. It was revealed that patients fight with nurses because of swearing at them, which results in a riot (Teffo & Rispel 2020).

### Health practitioners do not keep the confidentiality of youth accessing health facilities for pregnancy termination

The findings reveal that health practitioners do not keep the confidentiality of youth accessing health facilities for pregnancy termination. Concurred findings reveal that health practitioners who register youth during consultations also gossip about youth after consultations (Moon et al. 2023). Hence, these actions violate the rights of the patient and the *Department of Health POPIA Act*. This type of behaviour and attitude discourages youth from consulting in health facilities (Ralph & Hasselbacher 2023). The reluctance of unprofessional health workers portrays a negative health impact that forces youth not to consult their local clinics, and it leads them to patronise quacks for information regarding the termination of pregnancy (Teffo & Rispel 2020).

### Language is a barrier when healthcare professionals give instruction regarding the termination of pregnancy

The findings reveal that language is a barrier when healthcare professionals give instructions regarding the termination of pregnancy. A study conducted by Mavuso (2017) concurred with the findings that patient understands instruction better when it is provided in their home language. Contrary findings reveal that participants understood instruction given in other languages (Mbona, Kistan & Sebitloane 2023). Coinciding findings by Moon et al. (2023), who alluded that patients should provide diaries to take instruction notes in the hospitals. Mavuso (2017) attested that any patient must be accompanied by their trusted relative to ensure that they understand instructions given in the hospital either for medication or follow-up as well as referral.

### Health practitioners wanted consent from their parents before they terminated my pregnancy

The findings reveal that health practitioners wanted consent from their parents before they terminated my pregnancy. It was contended that a 12-year-old youth requires consent from parents for termination of pregnancy (Milford et al. 2023). However, contrary findings asserted that youth under the age of 12 must be given privacy in their consultation without any approval (Millar 2023). Youth development agency supported the findings by stating that youth under the age of 18 need consent from their parents or guardians (Mafa 2019). A study conducted in Zimbabwe reported that youth should be given the right to privacy when they visit hospitals for consultation regardless of age (Rugoho & Maphosa 2017). It implies that rejection of consultation from the hospital because of consent requests results in the loss of life (Millar 2023).

### Health practitioner informed their parent about the consultation they made regarding the termination of pregnancy

The findings reveal that the health practitioner informed their parent about the consultation they made regarding the termination of pregnancy. Concurred findings reveal that health practitioners violate the *POPIA Act* by sharing patient consultation (Kleinsmidt, Malope & Urban 2023). Similar findings indicate that health practitioners had a tendency to shout at youth in front of fellow colleagues and patients (Teffo & Rispel 2020). Furthermore, other health workers had a negative attitude towards youth, which resulted in a fear of visiting health facilities for consultations (Kleinsmidt et al. 2023). Coinciding findings reveal that parents are being informed about consultation either for contraceptives or termination of pregnancy by health workers (Mbona et al. 2023). A female participant indicated that she was ‘interrogated by her parent about why she visited a health facility without informing them’ (Rugoho & Maphosa 2017).

### Lack of pregnancy termination resources deterred youth from visiting health facilities

The findings reveal that the lack of pregnancy termination resources deterred youth from visiting health facilities. It was shown that termination of pregnancy is only performed at the hospital, while PHC facilities refer patients to the hospital (Moore et al. 2023). As a result, those who do not have money to visit hospitals tend to suffer, especially those who live in disadvantaged areas (Stifani et al. 2022). It was recommended that primary healthcare facilities should offer termination of services. However, only the community health centres are mandated to provide termination of pregnancy (Mbona et al. 2023). By so doing, it disadvantages those who are living far away from the facilities, while the nearest is the clinic that acts as a referral; this leaves youth devastated and results in births (Katz et al. 2022).

### The average waiting period at the public health facility is very long for pregnancy termination

The findings reveal that the average waiting period at the public health facility is very long for pregnancy termination. It was attested that this is because of a shortage of workers and a lack of resources in the public health facilities (Oda, Sento & Negera 2023). However, this led to poor services in the health facilities and caused burnout among youth in consultation queues. Coinciding findings reveal public health facilities mix patients of different diseases in an Outpatient Department (OPD) for consultation (Montero et al. 2023). As a result, youth wait too long, while the gynaecology section, which offers termination of pregnancy, is empty. Montero et al. (2023) alluded that when patients receive files, they must be allowed to go to the respective department for their consultations to avoid too much waiting period. This strategy will ensure that patients do not wait too long to provide for those who are living far from the health facilities (Oda et al. 2023).

## Conclusion

A multifaceted strategy is needed to address the problem of unplanned pregnancies among young people in the Vhembe area. Reduced access to contraceptives, thorough healthy sexual practices education programmes and open communication between parents and children are all essential elements. By effectively putting these suggestions into practice, healthcare professionals especially nurses, can arm youth with the information and resources they need to make informed decisions about their sexual health. Schools and community institutions should conduct comprehensive healthy sexual practices education. Topics, including sexually transmitted illnesses (STIs), contraception options and the value of consent, should all be included in this instruction. Young people can make educated decisions regarding their sexual health by being given proper information. It is necessary to expand access to contraception. Condoms, birth control tablets and long-acting reversible contraceptives (LARCs) should all be readily available in healthcare facilities and pharmacies. Efforts should also be made to lessen the stigma associated with the use of contraceptives so that young people are at ease obtaining these services. It is crucial to encourage honest communication between parents and their kids. For conversations about relationships and sexuality, parents should create a secure environment. Young people are more likely to seek advice from dependable adults than to engage in risky activities if an environment is created where inquiries may be expressed without fear of ridicule or judgement.

### Limitations of the study

In the province of Limpopo, one district municipality served as the study’s site. In addition, the study did not include the other four districts. Only data from students who were enrolled in school full time were obtained; dropout students were not included in the study.
